# Downregulation of nuclear STAT2 protein in the spinal dorsal horn is involved in neuropathic pain following chronic constriction injury of the rat sciatic nerve

**DOI:** 10.3389/fphar.2023.1069331

**Published:** 2023-01-18

**Authors:** Zhifeng Huang, Zijing Ding, Yangting Xu, Caiyun Xi, Liqiong He, Hui Luo, Qulian Guo, Changsheng Huang

**Affiliations:** ^1^ Department of Anesthesiology, Xiangya Hospital, Central South University, Changsha, China; ^2^ Xiangya School of Medicine, Central South University, Changsha, China; ^3^ National Clinical Research Center for Geriatric Disorders, Xiangya Hospital, Central South University, Changsha, China

**Keywords:** neuropathic pain, STAT2, spinal dorsal horn, proteomics, microglia

## Abstract

Regulation of gene transcription in the spinal dorsal horn (SDH) plays a critical role in the pathophysiology of neuropathic pain. In this study, we investigated whether the transcription factor STAT2 affects neuropathic pain and evaluated its possible mechanisms. A proteomic analysis showed that the nuclear fraction of STAT2 protein in the SDH was downregulated after chronic constriction injury of the rat sciatic nerve, which was associated with the development of neuropathic pain. Similarly, siRNA-induced downregulation of STAT2 in the SDH of naïve rats also resulted in pain hypersensitivity. Using RNA-sequencing analysis, we showed that reduction of nuclear STAT2 after chronic constriction injury was associated with increased expression of microglial activation markers, including the class II transactivator and major histocompatibility complex class II proteins. In addition, siRNA-induced downregulation of STAT2 promoted microglial activation and pro-inflammatory cytokine expression in the SDH. Taken together, these results showed that chronic constriction injury caused downregulation of nuclear STAT2 in the SDH, which may result in microglial activation and development of neuropathic pain. Our findings indicate that restoration of nuclear expression of STAT2 could be a potential pathway for the treatment of neuropathic pain.

## 1 Introduction

Pain is an evolutionary physiological protective mechanism in animals and is a warning signal of ongoing or impending tissue damage (Inoue and Tsuda, 2018). However, neuropathic pain caused by injury or disease of the somatosensory nervous system is a pathological condition that often persists and causes great distress to patients (Colloca et al., 2017). Because of the complex pathogenesis of neuropathic pain, current treatment options and medications often fail to achieve satisfactory results (Colloca et al., 2017). Therefore, exploring the pathogenesis of neuropathic pain and developing effective therapeutic drugs are of great importance for improvement of human health.

Peripheral nerve injury can induce transcriptional changes in pain-related genes in the dorsal root ganglion and spinal dorsal horn (SDH), which in turn lead to the development and maintenance of neuropathic pain (Li et al., 2017; Shen et al., 2019). A key step in the transcriptional regulation of pain-related genes is the import or export of transcriptional regulators into or from the nucleus (Marvaldi et al., 2020). Previous studies have found that many transcriptional regulators accumulate in the nucleus of neurons or glia after nerve injury, thus contributing to the onset of neuropathic pain. For example, nerve injury causes NF-κB to accumulate in the nuclei of SDH cells (Gao et al., 2020), which promotes the transcription of pro-inflammatory genes and the development of neuropathic pain. Nerve injury accelerates the retrograde transport of axonal signal transducer and activator of transcription 3 (STAT3) to the nucleus, which also facilitates transcription of inflammatory genes and the occurrence of neuropathic pain (Sheu et al., 2000; Hu et al., 2020). In contrast to these transcriptional regulators that exert pro-pain effects, some transcriptional regulators regulate pain-repressing genes, and nerve injury leads to a reduction in their distribution within the nucleus (Litke et al., 2022). Knowledge regarding transcriptional regulators that may exert analgesic effects is still limited.

In this study, we first used proteomic analysis to examine the nuclear fraction of proteins in the SDH in rats subjected to chronic constriction injury (CCI) of the sciatic nerve and sham-operated rats. We found that STAT2 expression is reduced in SDH nuclei after CCI. STAT2 has been reported to regulate the expression of major histocompatibility complex class II (MHC II) proteins (Zhao et al., 2007) and play an essential role in immune responses to extracellular and intracellular stimuli, driving the transcription of pro-inflammatory genes (Nan et al., 2018). We further showed that reduction of the nuclear distribution of STAT2 might lead to microglial activation and an increase in MHC II protein in the SDH. Our study indicates that restoration of the nuclear expression of STAT2 could be a potential treatment for neuropathic pain.

## 2 Materials and methods

### 2.1 Animals

Adult male Sprague-Dawley rats (Hunan SLAC Laboratory Animal Co., Ltd., Changsha, China) weighing 220–250 g were used for all experiments. All animals were raised in an environment with a 12-h light/dark cycle, free access to food and water, and appropriate temperature and humidity conditions for growth. The experiments were conducted strictly according to the National Institutes of Health Guide for the Protection and Use of Laboratory Animals and were approved by the Institutional Ethics Committee of Central South University (protocol code 2020sydw0445).

### 2.2 Rat model of CCI

Rats were adequately anesthetized with 3% pentobarbital sodium (50 mg/kg), and the CCI model was established according to the procedure described by Bennett and Xie (1988). Briefly, the skin was incised at the left sciatic nodule, and the muscles of each muscle layer were bluntly separated to expose the left sciatic nerve. Then, four snug ligatures (4-0) were tied to the sciatic nerve with equal tightness at a consistent interval. After ligation, the nerve was returned to the intermuscular space, and the skin wound was sutured. The sham-operated group underwent the same procedure, except for ligation of the sciatic nerve.

### 2.3 Behavioral assessment

To measure the paw withdrawal mechanical threshold (PWMT), rats were placed in a plastic cage with a metal mesh floor, and Von Frey filaments (North Coast Medical, San Jose, CA, United States) ranging from .4 to 26 g were applied vertically to the plantar surface of the left hind paw until the filament was slightly bent, as described in our previous studies (Shen et al., 2020; Zhang et al., 2022). The paw withdrawal threshold was determined using the up-and-down method. The PWMT was recorded as the minimum stimulus force required to induce withdrawal responses (lifting or licking).

We used a thermal pain test instrument (Tes7370, Ugo Basile, Comerio, Italy) to test the paw withdrawal thermal latency (PWTL) (Shen et al., 2020; Zhang et al., 2022). Briefly, rats were placed in the same chamber, which was placed on a 2-mm-thick heat-conducting glass plate. The rats were irradiated with a radiant heat source stimulator (cut-off time: 30 s) on the plantar surface of the left hind paw, and the thermal withdrawal latency was recorded. We repeated the measurement three times at 5-min intervals, and the mean latency was calculated as the PWTL.

The PWMT and PWTL of rats were measured 1 day before and 1–7 days after surgery, respectively. All behavioral tests were performed after the rats were acclimated to a specific individual chamber for at least 30 min.

### 2.4 Intraspinal injection

Bilateral injection of small interfering RNA (siRNA) into the spinal cord parenchyma results in spatially and temporally constrained stable gene transduction without persistent inflammation, tissue damage, or glial scar formation (Simonetti et al., 2013; Litke et al., 2022). After rats were fully anesthetized, the T13 vertebral body was traced by palpating the most caudal rib to determine the location of the incision. Then, the skin was incised to separate the subcutaneous connective tissue and muscle and fully expose the T12-L1 vertebral plate. A small amount of the lamina was removed at the junction of the lower edge of the T13 and the upper edge of the L1 vertebral body with occlusal forceps, working carefully and gently to avoid injury to the spinal cord (Chen et al., 2019). Using a stereotaxic instrument and a microinjection device, siRNA was injected into the dorsal horn of the lumbar expanded segment of the spinal cord of rats using a glass pipette needle at .8 mm on the left side of the midline of the spinal cord with an insertion depth of .6 mm. After injection, the procedure was paused to allow equilibration (approximately 1–2 min) before slowly withdrawing the glass needle. Finally, the wound was sutured, and the rat regained consciousness. The Stat2 (si-STAT2) and corresponding negative control (si-NC) siRNAs were designed and synthesized by RiboBio Co., Ltd. (Guangzhou, China).

### 2.5 Nuclear protein extraction and digestion

A sample was harvested for nuclear protein extraction using a Minute Cytosolic and Nuclear Extraction Kit (Invent Biotechnologies, Inc.) according to the manufacturer’s instructions. Briefly, 250 μl of cold extract A was added to each 20–30 mg sample, which was then homogenized with a tissue homogenizer until no solids were visible. Then, the sample was centrifuged for 5 min at 4°C and 14,000 × g, and the precipitate was retained. Next, .5 ml of buffer B was added, and the pellet was resuspended by vortexing for 10–20 s. The supernatant was completely removed after incubation on ice for 5 min and centrifugation at 2,000 × g for 2 min. The supernatant was aspirated into another pre-chilled clean centrifuge tube to obtain the nucleoproteins.

For digestion, protein samples were diluted by adding 100 mM triethylammonium bicarbonate to a urea concentration of less than 2 M after reduction and alkylation, and the samples were digested with trypsin in two stages (first stage, 1:50 trypsin/protein mass ratio, overnight; second stage, 1:100 mass ratio, 4 h).

### 2.6 Tandem mass tag labeling and high-performance liquid chromatography fractionation

After trypsin digestion, the peptides were desalted using a Strata X C18 SPE column (Phenomenex) and vacuum dried. The peptides were reconstituted in .5 M triethylammonium bicarbonate and processed according to the manufacturer’s protocol for the tandem mass tag kit. Briefly, one unit of tandem mass tag reagent was thawed and reconstituted in acetonitrile. The peptide mixtures were then incubated for 2 h, pooled, desalted, and dried by vacuum centrifugation.

An Agilent 300 Extend C18 column (5 μm particles, 4.6 mm ID, 250 mm length) was used to perform high-pH reversed-phase high performance liquid chromatography to fractionate the samples. Briefly, the peptides were first separated into 60 fractions over 60 min using a gradient of 8%–32% acetonitrile. The peptides were then combined into 18 fractions and dried by vacuum centrifugation.

### 2.7 Liquid chromatography tandem mass spectrometry analysis

The tryptic peptides were dissolved and directly loaded onto a homemade reversed-phase analytical column (15 cm in length, 75 μm ID). The gradient of solvent B began at 6% and was increased to 23% over 26 min, 23%–35% over 8 min, and 80% over 3 min and then was held at 80% for the final 3 min, all at a constant flow rate of 400 nl/min on an EASY-nLC 1000 UPLC system. The peptides were subjected to an NSI source followed by tandem mass spectrometry on a Q Exactive™ Plus (Thermo) instrument coupled online to the UPLC system. The m/z scan range was 350–1,800 for the full scan, and intact peptides were detected in the Orbitrap at a resolution of 70,000 with an electrospray voltage set at 2.0 kV. The peptides were then selected for tandem mass spectrometry using an NCE setting of 28 and resolution of 17,500.

### 2.8 Protein-protein interaction (PPI) analysis

PPI is defined as physical contact between two or more proteins. A PPI network is visualized as a graph for which every node represents a protein, and the edges indicate physical or functional interactions between proteins. PPI network analysis was performed using STRING online software (http://string-db.org/). The protein interaction information in the STRING database includes experimental results, computational predictions, and previous literature. Next, interaction data in tsv format were downloaded and then analyzed using Cytoscape (Version: 3.7.1) in a JAVA platform.

### 2.9 Real-time quantitative PCR (RT-qPCR)

To detect changes in gene expression levels, the affected lumbar segments of the rat spinal cord were collected for RT-qPCR. Total RNA was extracted using the TransZol Up kit (TransGen) according to the instructions, followed by reverse transcription and synthesis of the corresponding cDNA using the TransScript First-Step RT-PCR SuperMix kit (TransGen). RT-qPCR was carried out using an ABI Prism 7300 PCR system. The reaction conditions consisted of pre-denaturing at 94°C for 30 s, followed by 40 cycles of 94°C for 5 s and 60°C for 30 s. The relative expression of target genes was compared with that of GAPDH, as an endogenous reference gene, and calculated using the ΔΔCt method. The specific STAT2 primer was obtained from Sino Biological, and the other primers included ACTIN 5′- TGC​TAT​GTT​GCC​CTA​GAC​TTC​G-3′ (F) and 5′-GTT​GGC​ATG​AGG​TCT​TTA​C-GG-3′ (R); RT1-Ba 5′-AGA​AAC​AGC​AAG​CCA​GTC-3′ (F) and 5′-GGA​TGA​AGG​TGA​G-GTA​AGC-3′ (R); and class II transactivator (CIITA) 5′-CAT​ACT​CTG​TGT​GCC​ACC​ATG​G-3′ (F) and 5′-AGT​TCG​AT-CTC​TTC​CTC​CCC​A-3′ (R).

### 2.10 Western blot

Collected spinal cord tissues were lysed in radio immunoprecipitation assay lysis buffer (NCM Biotech) containing protease and phosphatase inhibitors. After ultrasonic homogenization, proteins were extracted by centrifugation, and the protein concentration was determined using a bicinchoninic acid assay. Following separation by 8% sodium dodecyl-sulfate polyacrylamide gel electrophoresis, protein samples were transferred to polyvinylidene difluoride membranes. The membranes were blocked in NcmBlot blocking buffer (NCM Biotech) for 15 min and subsequently incubated with primary antibodies as follows (4°C, overnight): rabbit anti-STAT2 (1:500, HuaBio), mouse anti-GAPDH (1:5000, ZENBIO), and mouse anti-H3 (1:1000, Cell Signaling Technology). The polyvinylidene difluoride membranes were washed with TBST buffer, followed by incubation with the corresponding secondary mouse or rabbit antibody (1:5000, goat anti-mouse, goat anti-rabbit, ZENBIO) for 2 h at room temperature. The membranes were developed using a ChemiDoc XRS System and Image Lab software (Bio-Rad, Universal Hood III, United States).

### 2.11 Immunofluorescence and microscopy

After the rats were anesthetized with sodium pentobarbital, they were perfused with phosphate-buffered saline solution and 4% paraformaldehyde. Immediately after perfusion, the rat spinal cord was removed and placed in test tubes pre-filled with paraformaldehyde for 12 h. The tissue was then treated sequentially with 15% and 30% sucrose and water for dehydration. The lumbar expansion segment of the rat spinal cord was cut into 10-µm frozen sections using a cryostat (Leica CM1860 cryostat, Germany). Next, the sections were blocked with an Immunol Staining Blocking Buffer (Beyotime, Shanghai, China) for 15 min at room temperature on a rocking platform and then washed with phosphate-buffered saline. Then, the sections were incubated with primary antibodies as follows (4°C, overnight): rabbit anti-STAT2 (1:100, ImmunoWay Biotechnology), mouse anti-NeuN (1:400, Novus), mouse anti-IBA1 (1:100, Abcam) and mouse anti-GFAP (1:300, Cell Signaling Technology). The next day, after washing with phosphate-buffered saline, the sections were incubated separately with the corresponding secondary antibodies (1:200, goat anti-mouse, goat anti-rabbit, Abcam) at room temperature for 1 h. Finally, staining was observed under a Leica DM5000B microscope (Leica Biosystems, Wetzlar, Germany).

### 2.12 Cell culture and transfection

The rat microglia cell line HAPI (Huiying biological Technology CO., Ltd., Shanghai, China) was cultured in Dulbecco’s modified eagle medium supplemented with 10% fetal bovine serum and 1% penicillin and streptomycin. The cells were seeded in six-well plates (2 × 10^5^ cells/well) and cultured at 37°C in a 5% CO_2_ incubator overnight. The cells were stimulated with lipopolysaccharides (LPS) (1 μg/ml) for 12 h and treated with medium containing minocycline hydrochloride or interferon-α (IFNα) compared to mock-treated cultures. For transfection experiments, siRNA targeting Stat2 was transfected into HAPI cell lines by Lipofectamine™ 3,000 to silence *Stat2*, while the control cells were transfected with negative control siRNA.

### 2.13 Statistical analysis

Statistical analysis was performed using two-tailed unpaired Student’s t-test or Mann-Whitney test for comparison between two groups, and one-way or two-way ANOVA followed by Bonferroni’s multiple comparisons test for comparison among multiple groups of data with Prism 7 (GraphPad Software Inc., San Diego, CA, United States; RRID: SCR_002798). Gene Ontology (GO) or Kyoto Encyclopedia of Genes and Genomes (KEGG) categories were analyzed using a two-tailed Fisher’s exact test to determine the enrichment of differentially expressed proteins (DEPs) among all identified proteins. A value of *p* < .05 indicated a statistically significant difference.

## 3 Results

### 3.1 Proteomic analysis of nuclear protein indicated changes in the STAT-related signaling pathway after CCI

**FIGURE 1:**
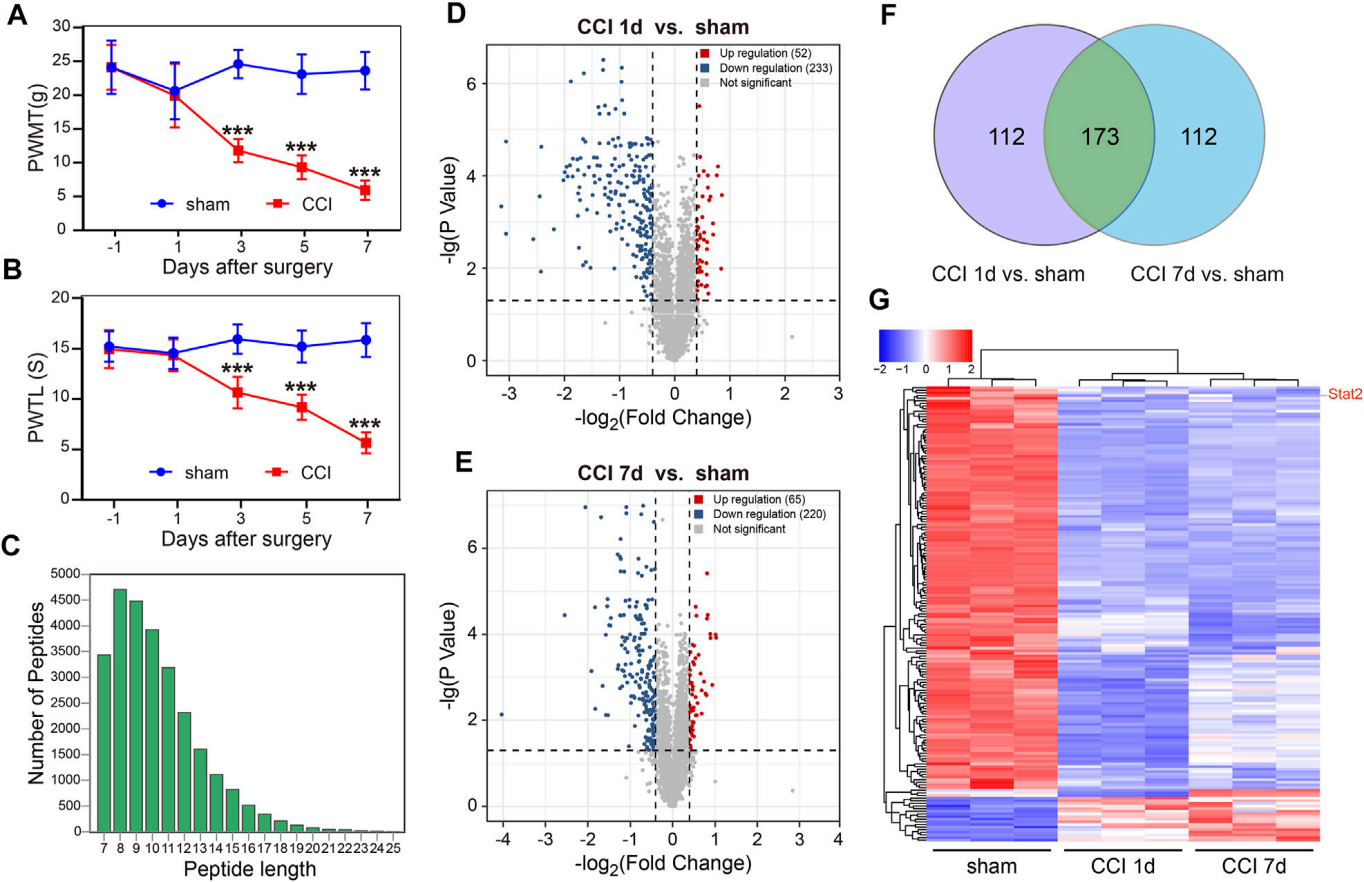
Global differentially expressed proteins (DEPs) in the spinal dorsal horn (SDH) of rats following chronic constriction injury (CCI). Rats developed neuropathic pain after CCI surgery, as evidenced by the decrease in the paw withdrawal mechanical threshold (PWMT) **(A)** and the paw withdrawal thermal latency (PWTL) **(B)** (*n* = 8, ***p < .001, two-way ANOVA with repeated measures followed by Bonferroni’s multiple comparisons test). **(C)** The quality control results of mass spectrometry data. The horizontal coordinate indicates the peptide length, and the vertical coordinate indicates the number of peptides. **(D)** A significant difference in nuclear proteins was found between the sham and CCI groups on day 1. **(E)** A significant difference in nuclear proteins was found between the sham and CCI groups on day 7. The horizontal coordinate represents the log2 value (Fold Change), and the vertical coordinate represents −lg (*p*-value). Upregulated genes are shown in red, downregulated genes are shown in blue, and genes with no significant differences are shown in gray. **(F)** The unique or shared DEPs in each of the paired groups. **(G)** The differences in 173 nuclear proteins at 1 and 7 days after CCI compared with those in the sham group.

We used the CCI model to induce neuropathic pain in rats and measured the pain behavior according to the PWMT and PWTL. Compared with sham-operated rats, the PWMT (*p* < .001, *n* = 8, Figure 1A) and PWTL (*p* < .001, *n* = 8, Figure 1B) were significantly decreased in CCI rats from days 3–7 after surgery. These results indicated that the neuropathic pain model had been successfully established.

Subcellular proteomic analysis was carried out on the nuclear fraction of the SDH in sham-operated rats (*n* = 3) and in rats 1 and 7 days after CCI (*n* = 3 at each time point). The quality control results of the mass spectrometry data showed that most peptide lengths were between 8 and 20 amino acid residues, which is in accordance with the pattern of trypsin digestion of peptides, indicating that the sample preparation met the standard (Figure 1C).

A total of 5,039 DEPs were identified, 4,469 of which contained quantitative information. Among all proteins measured, DEPs were defined as having a fold change of more than 1.3 and *p*-value < .05 in a *t*-test. Compared with the sham group, 52 nuclear proteins were upregulated and 233 nuclear proteins were downregulated on day 1 after CCI surgery (Figure 1D). 65 nuclear proteins were upregulated and 220 nuclear proteins were downregulated on day 7 after CCI surgery (Figure 1E). We also analyzed the unique or shared genes between each paired population and used a Venn diagram to represent the number of DEPs (Figure 1F). Compared with the sham group, 112 nuclear proteins were unique, and 173 nuclear proteins were shared in each group on day 1 and day 7 after CCI surgery. Among 173 DEPs, 153 nuclear proteins were downregulated and 17 nuclear proteins were upregulated in CCI rats on days 1–7, and 3 proteins were downregulated in CCI rats at 1 day and upregulated in CCI rats at 7 days after surgery (Figure 1G). The up- and downregulated DEPs at each time point and the detailed information regarding changes are listed in Supplementary Table S1.

### 3.2 Bioinformatics analysis suggested that STAT-related signaling pathways regulate neuropathic pain

**FIGURE 2:**
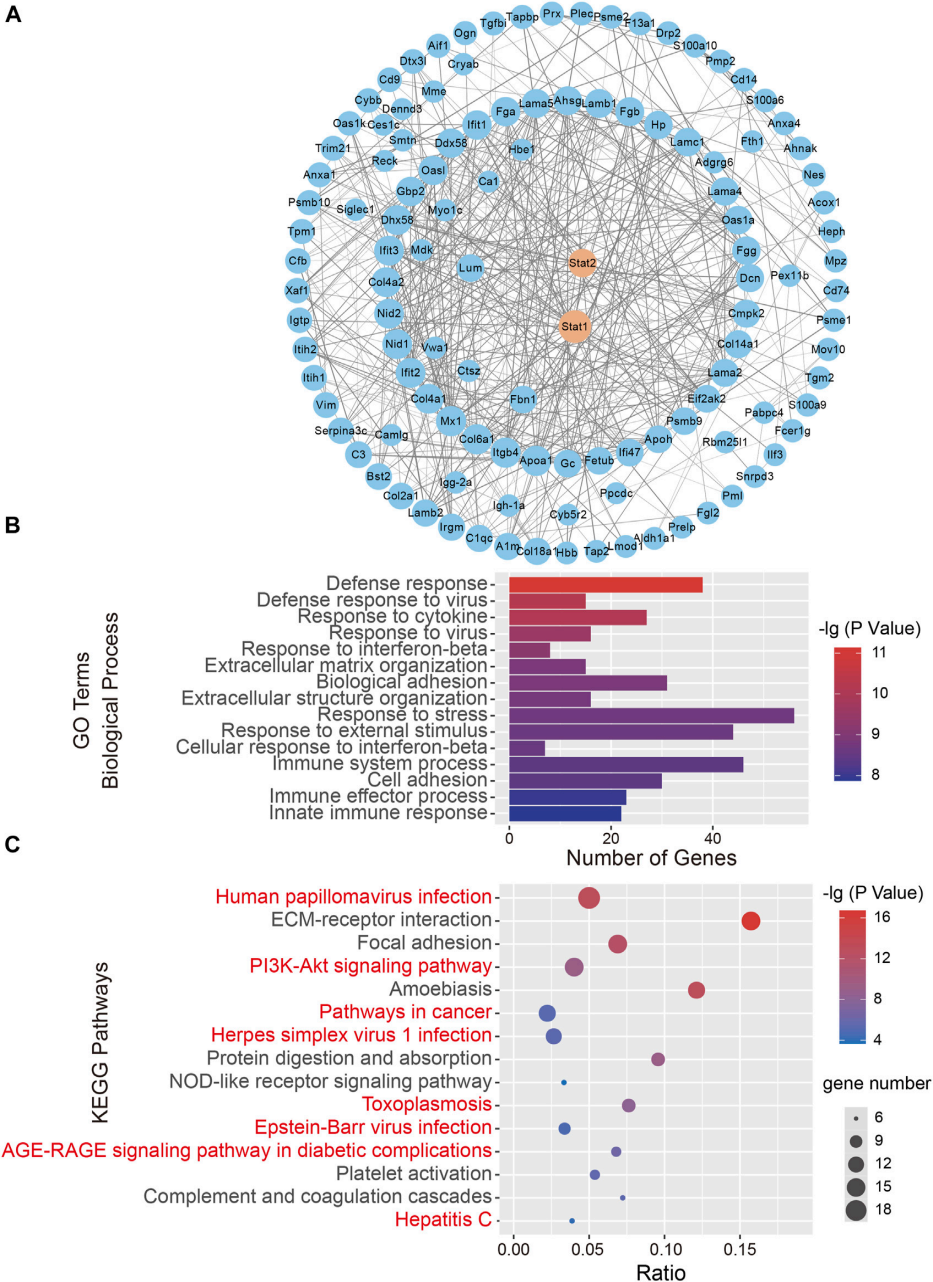
Bioinformatics analysis of 173 differentially expressed proteins (DEPs). **(A)** A protein-protein interaction (PPI) network analysis of DEPs. Network nodes represent proteins. Edges represent protein-protein associations. PPI enrichment *p* < .001. **(B)** The top 15 biological processes in Gene Ontology (GO) analysis. **(C)** The top 15 significantly enriched Kyoto Encyclopedia of Genes and Genomes (KEGG) pathways. The KEGG terms are plotted on the ordinate, and the enrichment factor is plotted on the abscissa. The size of the dots represents the gene number. The KEGG terms marked in red indicate eight signaling pathways directly related to STAT signaling pathways.

The consistent changes in 173 DEPs in rats at 1 and 7 days after CCI suggested that these proteins are likely to be involved in the occurrence and development of neuropathic pain. Therefore, we performed PPI network analysis to investigate the underlying relationships among the DEPs. The PPI network analysis revealed complex interactions among proteins, and most DEPs were highly associated with STAT1 and STAT2 (Figure 2A). Meanwhile, we used the Centiscape plugin (using three parameters: closeness, betweenness, and degree) in Cytoscape to screen key proteins in the PPI network map (Scardoni et al., 2009). The screening results also included the STAT1 and STAT2 proteins (Table 1).

**TABLE 1:** The central proteins in the PPI network screened using CentiScaPe analysis.

Protein name	Closeness	Betweenness	Degree
STAT1	**.003559**	**2699.168**	**20**
STAT2	**.003322**	**1552.583**	**18**
FGA	.003279	377.8293	17
LAMA5	.003215	1449.956	16
AHAG	.003175	316.4212	17
GBP2	.003165	1438.332	18
DCN	.003145	369.3456	13
NID1	.003125	398.4397	17
HP	.003106	284.2264	15

The bold values represent the top-ranked core proteins filtered by CentiScaPe analysis. Bold is added to emphasize the high rank of STAT family proteins.

To further characterize the 173 DEPs, GO and KEGG pathway enrichment analyses were performed on the dysregulated nuclear proteins. The GO analysis revealed that Biological Processes were enriched, including “Defense response,” “Response to cytokine,” “Defense response to virus,” and “Response to interferon-beta,” which indicated that immune and defense responses occurred in the SDH after CCI surgery (Figure 2B). Regarding the biological function enrichment in the GO analysis, we found that STAT protein family-related functions were obviously enriched. The KEGG analysis indicated that the main signaling pathways associated with DEP enrichment included “Human papillomavirus infection,” “PI3K-Akt signaling pathway,” and “Pathways in cancer”. We found that STAT-related signaling pathways were closely related to 8 of the top 15 KEGG pathways, such as “Pathways in cancer” (Verhoeven et al., 2020), “Hepatitis C” (Heim et al., 2016) and “Epstein-Barr virus infection” (Jangra et al., 2021) (Figure 2C). Taken together, these findings reveal that the STAT protein-related signaling pathway could be involved in the development of neuropathic pain.

### 3.3 CCI led to a reduction of STAT2 protein in SDH nuclei

**FIGURE 3:**
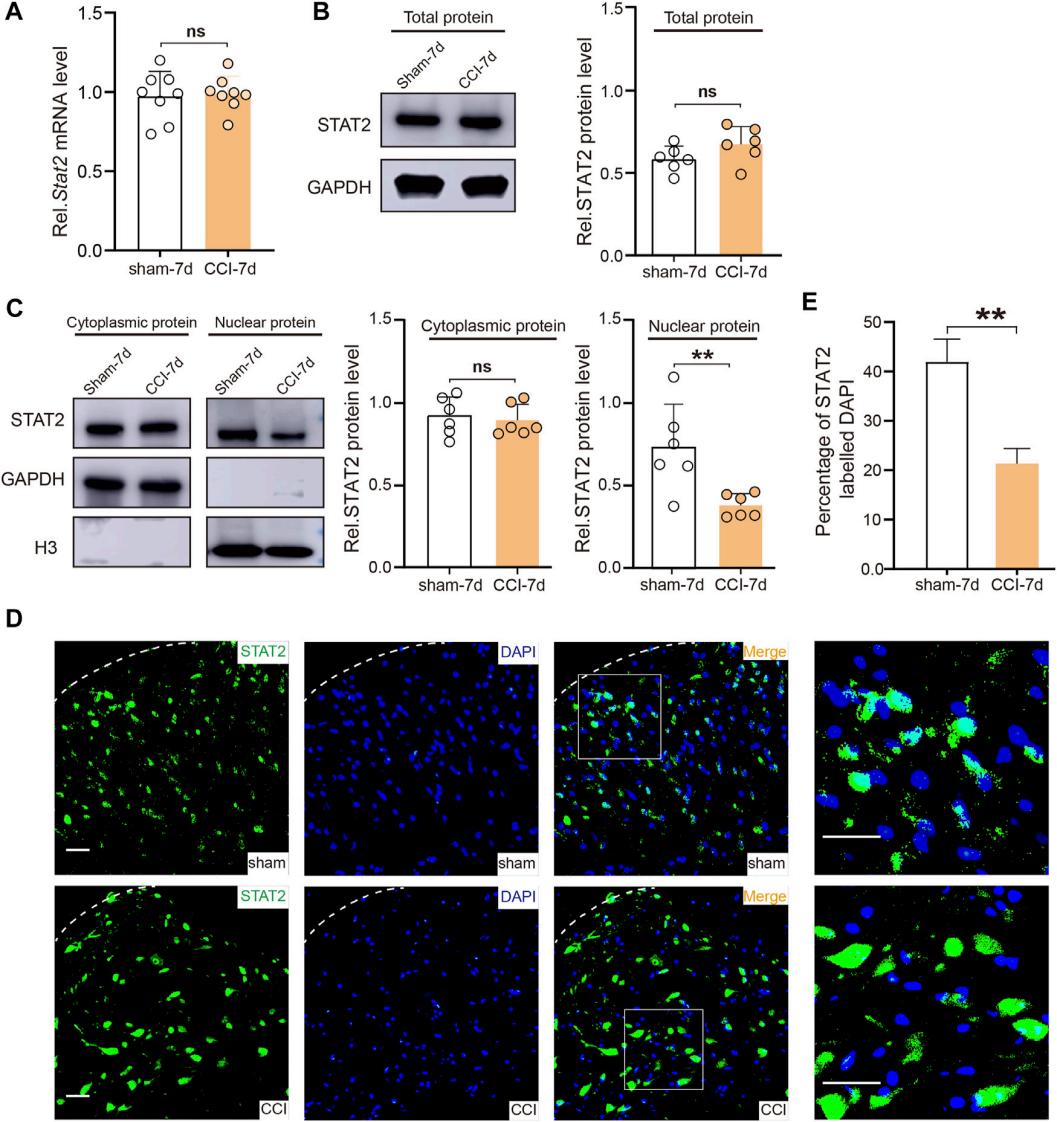
Chronic constriction injury (CCI) downregulated STAT2 in spinal dorsal horn (SDH) nuclei. **(A)** The mRNA expression of Stat2 in the SDH was not altered after CCI surgery (*n* = 8, two-tailed unpaired Student’s t-test). **(B)** STAT2 protein expression in the SDH of the CCI group was not different from that in the sham group (protein expression was normalized to the GAPDH level) (*n* = 6, two-tailed unpaired Student’s t-test). **(C)** STAT2 protein expression in the SDH of the CCI group was decreased in the nuclear fraction (protein expression was normalized to the H3 level) but not different from in the cytoplasmic fraction (protein expression was normalized to the GAPDH level) compared to the sham group (*n* = 6, **p < .01, two-tailed unpaired Student’s t-test). **(D)** Immunofluorescence staining of STAT2 (green) and DAPI (blue) in the injured ipsilateral SDH on day 7 after sham or CCI surgery (scale bar = 50 µm). **(E)** The proportion of STAT2 co-localized with DAPI (nuclear marker) was decreased in the ipsilateral SDH after CCI surgery (compared with sham-operated rats, **p < .01, *n* = 3, two-tailed unpaired Student’s t-test).

The relationship between STAT1 and pain has been intensively investigated in previous studies (Busch-Dienstfertig et al., 2012; Song et al., 2017; Wu et al., 2022). However, it is still unclear whether STAT2 also plays a role in neuropathic pain. We found that CCI surgery did not change the mRNA and total protein expression of STAT2 in the SDH (Figures 3A, B). However, consistent with the proteomic results, the western blot analysis showed that nuclear expression of STAT2 in the SDH was downregulated after CCI (Figure 3C). Furthermore, the immunofluorescence analysis also showed that STAT2 colocalization in the nucleus was reduced after CCI surgery (Figures 3D, E). These results indicate that CCI leads to a reduction of STAT2 protein in SDH nuclei.

### 3.4 Downregulation of STAT2 induced nociceptive hypersensitivity in naïve rats

**FIGURE 4:**
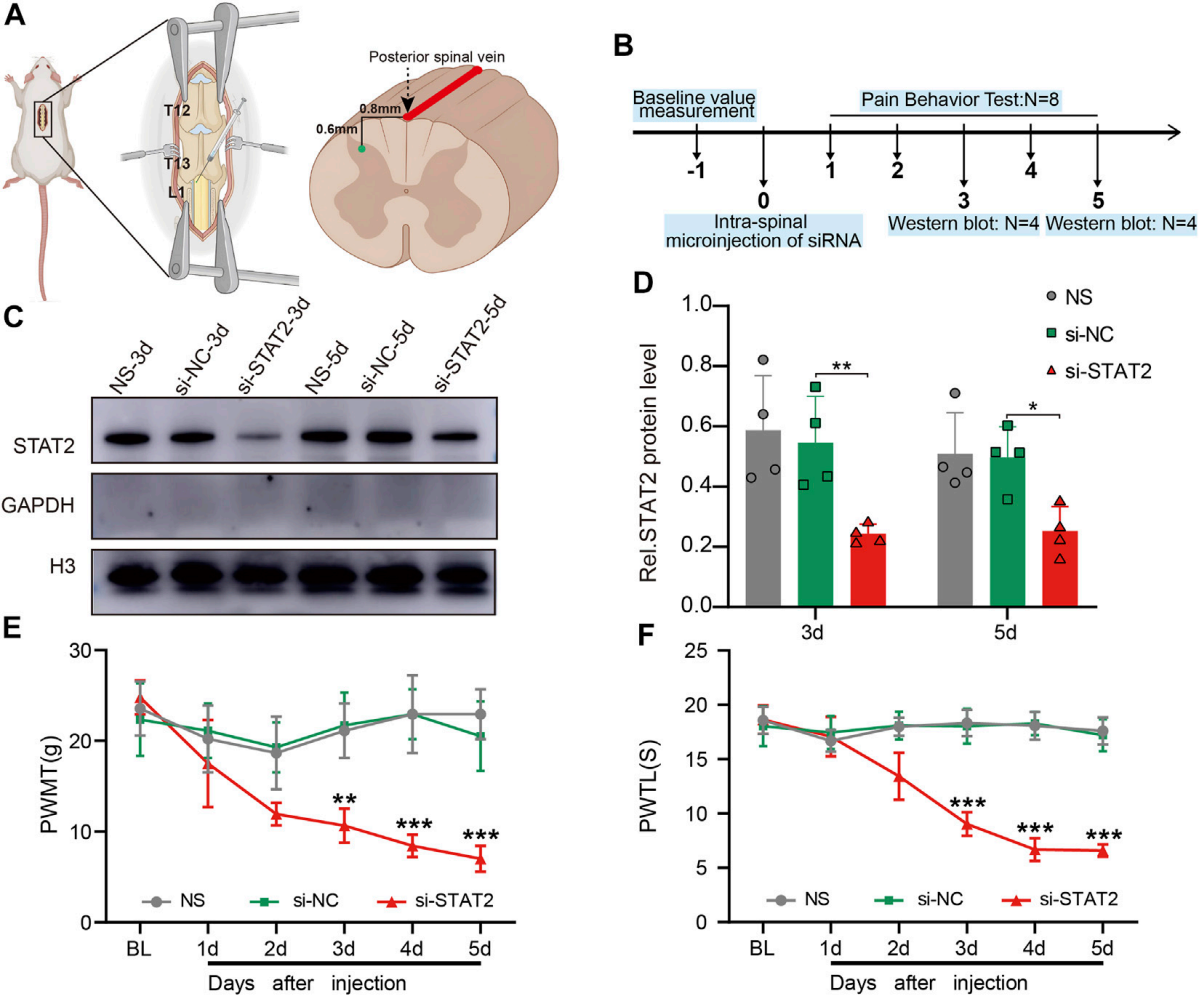
Downregulation of STAT2 induced nociceptive hypersensitivity. **(A)** The specific location of the intraspinal injection of si-STAT2. **(B)** The time points of si-STAT2 administration in naïve rats. **(C,D)** Nuclear expression of STAT2 was reduced in the spinal dorsal horn (SDH) after si-STAT2 injection compared with those in rats treated with normal saline (NS) or the corresponding negative control (si-NC) (*n* = 4, **p < .01, *p < .05, one-way ANOVA followed by Bonferroni’s multiple comparisons test). **(E,F)** Injection of si-STAT2 decreased the paw withdrawal mechanical threshold (PWMT) **(E)** and paw withdrawal thermal latency (PWTL) **(F)** in naïve rats from days 3–5 (si-STAT2 vs. si-NC, *n* = 8, **p < .01, ***p < .001, two-way ANOVA with repeated measures followed by Bonferroni’s multiple comparisons test).

To further understand the function of STAT2 in neuropathic pain, we microinjected si-STAT2 into the SDH of naïve rats, thereby reducing STAT2 protein expression in the SDH. The intraspinal injection was administered in the T3-L1 segment of the spinal cord, .8 mm from the left side of the posterior spinal vein at a depth of .6 mm (Figures 4A, B). The western blot results showed that STAT2 protein was significantly decreased in nuclei on days 3 and 5 after si-STAT2 injection compared with those from rats treated with normal saline and si-NC injection (Figures 4C, D). The behavioral analysis showed that si-STAT2 caused a dramatic decrease in the PWMT and PWTL from days 3–5 after treatment (Figures 4E, F). These results suggest that downregulation of STAT2 protein leads to nociceptive hypersensitivity.

### 3.5 Downregulation of STAT2 was associated with increased microglial activation marker expression

**FIGURE 5:**
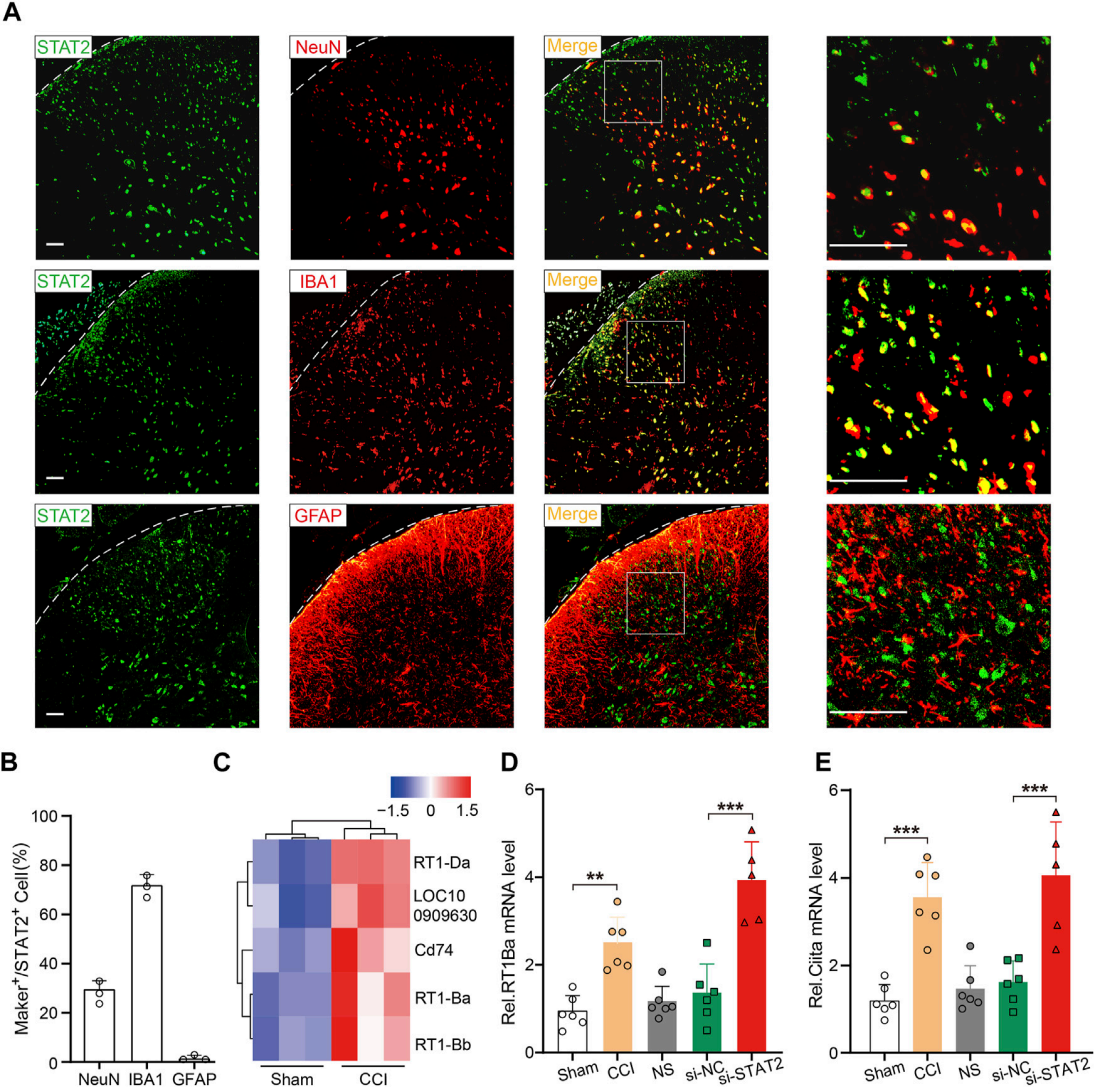
Downregulation of STAT2 increased expression of microglial activation markers. **(A)** Representative images showing immunoreactivity for STAT2 (green) double labeled with NeuN-labeled neurons (red), IBA1-labeled microglia (red) or GFAP-labeled astrocytes (red) (scale bar = 50 µm). **(B)** The percentage of marker-positive (NeuN, IBA1, and GFAP) (green) cells relative to STAT2-positive (red) cells (*n* = 3). **(C)** An RNA sequencing analysis heat map shows that major histocompatibility complex class II-associated genes were significantly upregulated in the SDH of chronic constriction injury (CCI) rats compared with those in the sham group. **(D,E)** Expression of RT1-Ba and Ciita mRNA was increased after CCI surgery and si-STAT2 injection (*n* = 6/5 rats/group, **p < .01, one-way ANOVA followed by Bonferroni’s multiple comparisons test).

STAT2 is involved in regulating MHC II expression, a marker of microglial activation (Sweitzer and DeLeo, 2002; Zhao et al., 2007; Dominguez et al., 2008). Consistently, we found that STAT2 was mainly co-localized with IBA1 (microglia biomarker) in the SDH (Figures 5A, B). RNA sequencing of the rat SDH revealed that expression of MHC II-related genes (RT1-Ba, RT1-Bb, RT1-Da, Cd74) was significantly upregulated after CCI surgery (Figure 5C). The expression of RT1-Ba, which is involved in encoding the rat MHC II, was further detected by PCR, which showed that RT1-Ba was significantly upregulated in CCI rats and in rats that received si-STAT2 injection (Figure 5D). The mRNA expression of Ciita, which is a major MHC II regulator (Tosi et al., 2002), was also significantly increased after CCI surgery and si-STAT2 injection (Figure 5E). These results suggested that downregulation of STAT2 is associated with increased expression of microglial activation markers.

### 3.6 Downregulation of STAT2 promoted microglial activation and expression of pro-inflammatory cytokines

**FIGURE 6:**
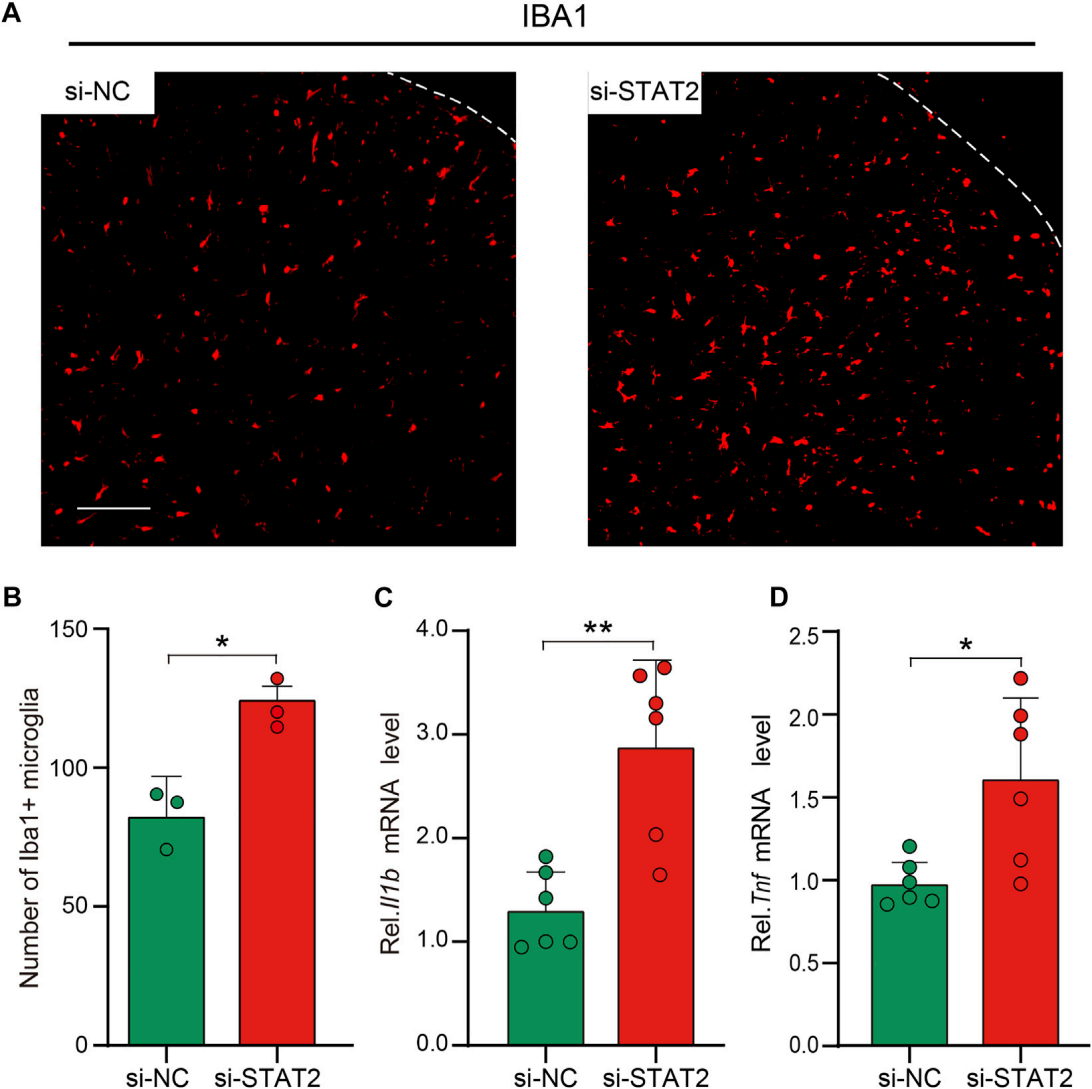
Downregulation of STAT2 promoted microglial activation and Tnf and Il1b expression. **(A)** Negative control (si-NC) rats had resting microglia with a ramified morphology, whereas si-STAT2 rats had activated microglia with an amoeboid morphology. (*n* = 3, scale bar = 100 µm). **(B)** si-STAT2 treatment increased the number of microglia in the spinal dorsal horn (SDH) (*n* = 3, *p < .05, Mann-Whitney test). **(C,D)** Expression of Il1b and Tnf mRNA was significantly increased in the SDH after intraspinal injection of si-STAT2 (*n* = 6, **p < .01, *p < .05, two-tailed unpaired Student’s t-test).

Microglial activation in the SDH is crucial for the development of neuropathic pain after peripheral nerve injury (Gu et al., 2016; Zhao et al., 2017; Inoue and Tsuda, 2018). After si-STAT2 injection, the number of microglia in the SDH was increased, and the SDH exhibited an activated cellular morphology, with amoeboid cell bodies (Figures 6A, B). Expression of the pro-inflammatory genes Il1b and Tnf was also increased on day 5 after intraspinal injection of si-STAT2 (Figures 6C, D). These results demonstrated that downregulation of STAT2 promotes microglial activation and expression of pro-inflammatory cytokines.

### 3.7 STAT2 in the nuclei negatively regulates pro-inflammatory gene expression in microglia

**FIGURE 7:**
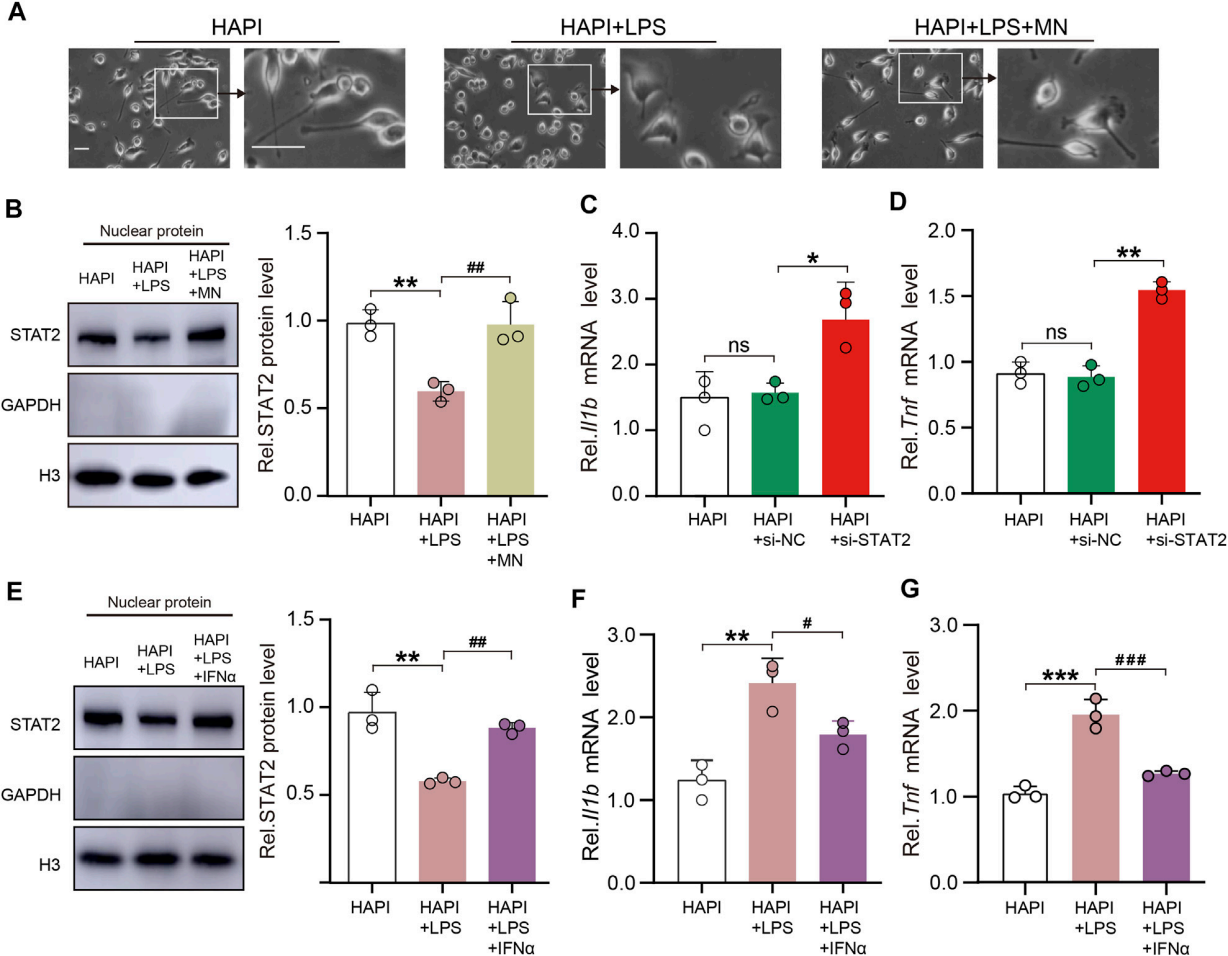
STAT2 in the nuclei negatively regulates pro-inflammatory gene expression in microglia. **(A)** Representative images of HAPI microglia showed that lipopolysaccharides (LPS) activated microglia and minocycline (MN) significantly inhibited microglia activation (Activated microglia had enlarged cytosomes, shortened protrusions, and amoeba-like cell morphology, scale bar = 50 µm). **(B)** Nuclear expression of STAT2 was reduced in activated HAPI microglia but it returned to basal level after the treatment of MN (HAPI vs. HAPI + LPS, **p < .01; HAPI + LPS vs. HAPI + LPS + MN, ^##^ *p* < .01, *n* = 3, one-way ANOVA followed by Bonferroni’s multiple comparisons test). **(C,D)** Expression of Il1b and Tnf mRNA was significantly increased in HAPI microglia after transfection of si-STAT2 (*n* = 3, *p < .05, **p < .01, one-way ANOVA followed by Bonferroni’s multiple comparisons test). **(E)** Nuclear expression of STAT2 was restored after treatment of IFNα (HAPI vs. HAPI + LPS, **p < .01; HAPI + LPS vs. HAPI + LPS + IFNα, ^##^ *p* < .01; *n* = 3, one-way ANOVA followed by Bonferroni’s multiple comparisons test). **(F,G)** Treatment of IFNα reversed the LPS-induced upregulation of Il1b and Tnf mRNA expression in HAPI microglia (HAPI vs. HAPI + LPS, **p < .01, ***p < .001; HAPI + LPS vs. HAPI + LPS + IFNα, ^#^ *p* < .05, ^###^ *p* < .001, *n* = 3, one-way ANOVA followed by Bonferroni’s multiple comparisons test).

To further evaluate the role of STAT2 in microglia, we used LPS as a stimulant of the microglial reactivity *in vitro* experiment. The morphology of HAPI cells showed that LPS successfully induced microglia activation (Figure 7A). The nuclear expression of STAT2 was significantly downregulated in activated microglia (Figure 7B). Interestingly, the nuclear expression of STAT2 was restored to basal levels after treatment of minocycline, an inhibitor of microglia (Kobayashi et al., 2013) (Figures 7A, B). Next, we assessed the impact of si-STAT2 transfection on the expression of pro-inflammatory in microglia. Consistent with the *in vivo* results, downregulation of STAT2 increased the expression of Il1b and Tnf in HAPI cells (Figures 7C, D).

It is well known that as a hallmark of type I interferons (e.g., IFNα) activation, STAT2 rapidly localizes to the nucleus and recognizes specific DNA targets upon IFNα stimulation (Banninger and Reich, 2004; Lee et al., 2020). To elucidate whether restoration of nuclear expression of STAT2 could attenuate the expression of pro-inflammatory factors, IFNα was used to facilitate the nuclear import of STAT2 *in vitro* experiments. Western blot analysis indicated that IFNα restored the nuclear expression of STAT2 in HAPI cells treated by LPS (Figure 7E). We then evaluated if the expression of pro-inflammatory factors were changed in activated HAPI cells exposed to IFNα. The results showed that LPS-induced upregulation of Il1b and Tnf were inhibited following the restoration of the nuclear distribution of STAT2 by IFNα (Figures 7F, G). Taken together, our data indicated that STAT2 in the nuclei negatively regulates pro-inflammatory gene expression in microglia.

## 4 Discussion

Our study demonstrates that a decrease in nuclear STAT2 is involved in the development of neuropathic pain. Using proteomic analysis, we investigated the nuclear fraction of the rat SDH after CCI and found that STAT2 was significantly downregulated. Downregulation of nuclear expression of STAT2 induced microglial activation in the SDH and pain hypersensitivity in rats. Restoration of the nuclear expression of STAT2 inhibited the expression of pro-inflammatory factors in activated microglia. These results suggest that downregulation of nuclear STAT2 protein in the SDH contributes to the development of neuropathic pain induced by peripheral nerve injury.

The bioinformatic analysis subsequent to the proteomics analysis revealed that the majority of DEPs in the SDH of CCI rats were highly associated with STAT1 and STAT2. STAT1 and STAT2 are important members of the STAT protein family, both essential for the classical host immune defense system. STAT1 plays a key role in chemokine production mediated by tumor necrosis factor-α and/or interferon-γ (Han et al., 2011; Jeong et al., 2015). In addition, numerous studies have revealed that STAT1 is involved in neuropathic pain *via* multiple pathways, including controlling NF-κB p65 nuclear translocation, increasing MHC II expression in microglia, and positively regulating P2Y14 receptors (Song et al., 2017; Guo et al., 2021; Wu et al., 2022). Recent reports indicate that STAT2, a widespread cytokine regulator, plays a role in inhibiting STAT1 in multiple signaling pathways (Ho et al., 2016). However, most studies on STAT2 have focused on host defense against viral infection or oncogenic effects, and few reports have demonstrated the involvement of STAT2 in neuropathic pain. Therefore, we focused on exploring the role of STAT2 in neuropathic pain in this study.

Our findings suggested that the downregulation of nuclear STAT2 protein in the SDH is involved in neuropathic pain following CCI surgery. However, questions remain regarding the mechanism that contributes to downregulation of nuclear STAT2 after CCI surgery. The nuclear distribution of STAT2 has been associated with its phosphorylation level. However, after nuclear import of phosphorylated STAT2, it redistributes to the cytoplasm after it is dephosphorylated in the nucleus (Banninger and Reich, 2004). Therefore, further studies are needed to detect the phosphorylation level of STAT2 after CCI to clarify its specific mechanism.

MHC II, a key molecule in inflammatory and autoimmune disease, is mainly expressed in microglia in the central nervous system and is a marker of microglial activation. Increasing evidence has shown that a high level of MHC II expression is present in the SDH after peripheral nerve injury and indicates that MHC II is an important molecule involved in chronic pain (Sato-Takeda et al., 2006; Dominguez et al., 2008; Zhou et al., 2019). STAT1 promotes the expression of MHC II, contributing to pain hypersensitivity (Song et al., 2017). Furthermore, the expression of MHC II is increased in STAT2-null macrophages (Zhao et al., 2007). Consistently, we observed that the expression of MHC II and its major transcriptional regulator, CIITA, was increased after CCI surgery and si-STAT2 injection. These findings suggested that STAT2 is involved in neuropathic pain by regulating MHC II expression after CCI. In contrast to other STAT proteins, STAT2 does not form a homodimer, but it forms a heterodimeric trimeric complex consisting of STAT2, STAT1, and interferon regulatory factor 9 to recognize DNA targets (Blaszczyk et al., 2016). Therefore, whether regulation of STAT1 phosphorylation is involved in the regulation of MHC II by STAT2 needs to be further explored.

STAT2 has been reported to provide a dominant benefit for proliferation of lung cancer cells (Nan et al., 2018). During the early stages of peripheral nerve injury, no obvious proliferation of neurons or astrocytes occurs in the spinal cord, and microglia are considered the main proliferating cells in response to peripheral nerve injury (Shen et al., 2019). Thus, the decreased nuclear distribution of STAT2 may be involved in proliferation signaling in microglia after CCI. Convincing evidence has established that microglial activation and pro-inflammatory cytokine release are important pathological features of neuropathic pain (Echeverry et al., 2017; Albrecht et al., 2018; Xia et al., 2021). Our study showed that microglia were activated after si-STAT2 injection, and the pro-inflammatory factors Il1b and Tnf were abundantly expressed. Consistent with the *in vivo* results, downregulation of STAT2 increased the expression of pro-inflammatory factors in microglia cell line. Thus, STAT2 might be involved in neuropathic pain through regulation of microglial activation in the SDH.

It has been reported that MHC II which is implicated in both cytokine and chemokine induction is required for microglial activation (Harms et al., 2013; Subbarayan et al., 2020). Furthermore, numerous studies have shown that pro-inflammatory factors such as IL1B and TNF can reactivate microglia in a positive feedback mechanism (Kuno et al., 2005; Block et al., 2007; Brás et al., 2020). Based on our results, STAT2 could be involved in microglial activation by increasing the expression of MHC II and pro-inflammatory factors, contributing to the pathogenesis of neuropathic pain. However, considering that the expression of STAT2 was also observed in a small portion of neurons in the SDH, the role of neuronal STAT2 in neuropathic pain could not be ruled out as a recent study indicates that STAT2 is also involved in the neuronal innate immune response (Monogue et al., 2022). Further studies are still needed to explore the cell-specific role of STAT2 in the pathogenesis of neuropathic pain.

In summary, this study demonstrated an important role of STAT2, presumably *via* regulating microglial activation, in the development of neuropathic pain after CCI. Downregulation of nuclear STAT2 contributed to microglial activation and the production of pro-inflammatory factors. Our study provided proof-of-concept data indicating that restoration of nuclear STAT2 expression in the SDH could be a potential therapeutic strategy for neuropathic pain.

## Data Availability

The datasets presented in this study can be found in online repositories. The names of the repository/repositories and accession number(s) can be found below: http://www.proteomexchange.org/, PXD034960.
